# New insight for metformin against bladder cancer

**DOI:** 10.1186/s41021-017-0074-z

**Published:** 2017-04-01

**Authors:** Amr Ahmed EL-Arabey

**Affiliations:** 1 Pharmacology and Toxicology Department, Faculty of Pharmacy, Al-Azhar University, Nasr City, Cairo, Egypt; 2 CAS-TWAS Fellowship at University of Science and Technology of China (USTC), Hefei, 23027 China

**Keywords:** Metformin, Bladder, Cancer, Therapy, New, Insight

## Abstract

International Agency for Research on Cancer (IARC) estimated that bladder cancer is the ninth most common cancer in the world, with 430,000 new cases and 165,000 deaths in 2012. Bladder cancer represents the fourth most common cancer in men and ninth most common cancer in women. It is the second most prevalent cancer in men 60 years of age or older in United States. Looking further down, continuing advancements in cancer research could potentially offer more choices for clinician and patient with longer survival and better quality of life. Although, bladder cancer represents an ideal tumor model to test and apply cancer prevention strategies; there are limited studies about application of metformin in the management of bladder cancer. Here, I will shed light on the proposed mechanisms of anti-carcinogenic effects of metformin and cohort of these mechanisms with the novel application of metformin as therapy of bladder cancer.

## Background

International Agency for Research on Cancer (IARC) announced that bladder cancer is the ninth most common cancer in the world, with 430,000 new cases and 165,000 deaths in 2012. Incidence of bladder cancer is predominantly high in males with 77% of the cases, sorting it the 7^th^ highest on incidence and 9^th^ highest on mortality. For females it is the 19^th^ highest on incidence and 17th highest on mortality. The incidence of bladder cancer is almost 3 times higher in more developed countries compared to less developed countries [1]. At initial diagnosis, about 75–80% of cases are Non-Muscle-Invasive Bladder Cancer (NMIBC), with the remaining Muscle- Invasive Bladder Cancer (MIBC) [2]. Transurethral Resection of Bladder Tumor is standard therapy for NMIBC combined with subsequent intravesical therapy, with a high five year survival rates. However, the five year progression and recurrence rates can add up to 1–45 and 31–78% respectively after the first treatment [3]. Thus, finding successful way of preventing NMIBC progression and recurrence is required urgently.

Several risk factors, such as smoking, paint and human papillomavirus infection, have been involved in carcinogenesis of urinary bladder [4]. A recent meta-analysis of 15 cohort studies concluded that pre obese has a significantly increased risk of bladder cancer by 7%, while obesity increases the risk of bladder cancer by approximately 10%. Moreover, the dose–response meta-analysis of this study shows a linear association between Body Mass Index (BMI) and bladder cancer, and displays each 5 kg/m^2^ increment of BMI matched to a 4.2% increase in risk of bladder cancer [5]. Furthermore, serum levels of insulin and IGF-1 which increased in obesity and/or metabolic disease patients may be facilitate tumorigenesis, proliferation, survival and appear to be a major mechanism linking obesity to cancer [6].

Diabetes mellitus (DM) was first investigated as a risk factor for cancer death at the beginning of the 20th century, when the etiologies of these two major deadly diseases

Diabetes mellitus (DM) was first investigated as a risk factor for cancer death at the beginning of the 20th century, when the etiologies of these two major deadly diseases were unknown. The fundamental burden of association between cancer and diabetes has been a motivation for researchers to look for prevention strategies that can simultaneously affect both diseases and reduce their overlapping load. Hence, a clarification of the association between DM and cancer is significant for disease prevention and management. Interestingly, several studies indicate that patients with diabetes have increased risk of several malignancies, including bladder, colon and rectum cancers. In 2013, meta-analysis study concluded that individuals with diabetes may have more than 35% increased risk of bladder cancer by comparison with non-diabetes individuals [7].

Metformin, a biguanide, was approved by the United States Food and Drug Administration in 1995 as an oral hypoglycemic agent. Given alone or in combination with a sulfonylurea, metformin improves glycemic control and lipid concentrations in patients who respond poorly to dietary control or to a sulfonylurea alone [8]. In 2005, the first attracted attention of metformin as promising drug to suppress not only serum glucose levels but also the incidence of various cancers in an observational study [8]. Moreover, metformin is commonly used off-label for metabolic syndrome [9] and treat weight gain induced by antipsychotic medications [10]^.^

Randomized controlled trials (RCTs) are widely admitted as the ‘gold standard’ for accumulating robust evidence for any health concern intervention. Recently, the European Institute of Oncology (IEO), Milan, Italy, was conducted a randomized, phase II, double-blind, placebo-controlled trial in women with stage I-IIa breast cancer candidates for elective surgery who received either metformin or placebo for 4 weeks before surgery. The authors concluded that a lower Ki-67 LI in ductal hyperplasia under metformin in women with abdominal obesity, the hallmark of insulin resistance, in line with cancer tissue. Thus, metformin selectively decreased Ki-67 in Human Epidermal Growth factor Receptor 2 (HER2)-positive cancers and in women with extra markers of insulin resistance [11]. Interestingly, recent study confirmed that patients with low Ki-67 expressions, negative epidermal growth factor receptor staining and preoperative positive urine cytology appear to be more sensitive to intravesical instillations for bladder recurrence prevention after radical nephroureterectomy [12].

**Fig. 1:**
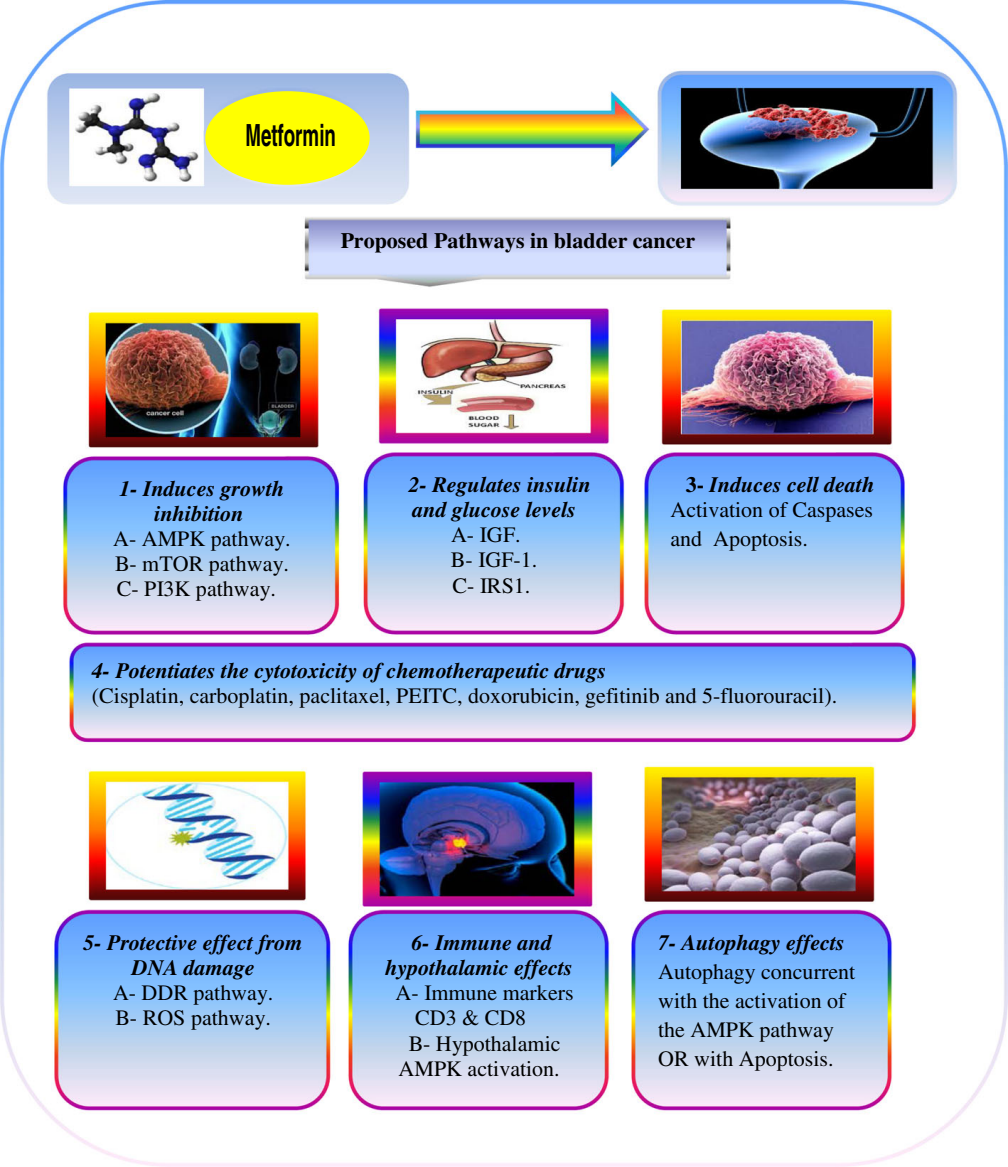
Proposed mechanisms of anti-carcinogenic effects of metformin against bladder cancer: (1) metformin induces growth inhibition, (2) metformin regulates insulin and glucose levels, (3) metformin induce cell death, (4) metformin potentiates the cytotoxicity of chemotherapeutic drugs, (5) metformin’s association with oxidative stress, DNA Damage Response (DDR), (6) immune and hypothalamic effects of metformin and (7) autophagy effects of metformin

Although, bladder cancer represents an ideal tumor model to test and apply cancer prevention strategies; there are limited studies about application of metformin in the management of bladder cancer. Furthermore, DM and obesity are considered as risk factors for bladder cancer. Metformin, a first-line oral anti-diabetic, has been demonstrated to prevent cancer and reduce cancer mortality among diabetic patients in observational studies [13]. Hence, in this review, I will shed light on the proposed mechanisms of anti-carcinogenic effects of metformin and correlation of these mechanisms with bladder cancer according to recent published literature (Fig. 1).

### Anti-carcinogenic effects of metformin

Various epidemiologic studies have shown that metformin is associated with reduced risk of cancer in diabetic patients [8, 14]. In addition, several studies have shown that the use of metformin significantly reduces the risk of cancers like breast [15], pancreatic [16] and prostate [17]. Interestingly, a recent meta-analyses/systematic reviews have examined the overall incidence of cancer in patients with diabetes taking metformin versus not and this study concluded that patients with diabetes who are treated with metformin have an approximately one-third reduction in the overall incidence of cancer [18]. Moreover, metformin has also been associated with lower overall cancer-related mortality [19]. Furthermore, the potential antitumor effects of metformin have been evaluated in numerous in vitro and in vitro studies on several cancer models including breast, endometrial, ovarian, pancreatic, lung, prostate, head and neck carcinomas, acute myeloid leukemia, glioma [20] and Colorectal cancer carcinogenesis (CRC) [21]. Clinically, the first notified trial of metformin for inhibiting CRC in humans provides preliminary evidence that metformin suppresses colonic epithelial proliferation and rectal aberrant crypt foci formation in humans, suggesting that metformin should be re-evaluated as promising drug for the chemoprevention of CRC [22].

Metformin can improve treatment outcomes in preclinical models of cancer, particularly in the obese setting and reduces the incidence of cancer in diabetic patients as well as improves survival in newly diagnosed cases. In addition, metformin has been shown to target cancer cells, transcription factors, microRNAs, DNA damage, cancer stem cells, and metabolism [6]. Recently, pioneer work by *Incio* et al.*,* [23]. proved that metformin in overweight/obese condition, reprograms the fibro-inflammatory tumor microenvironment and lately reduces metastasis in pancreatic cancer models [23].

### Metformin and bladder cancer

Study by Tseng, reported that metformin use is associated with a decreased risk of bladder cancer in Taiwanese patients with type-2 DM [24]. Moreover, a single-institution retrospective cohort study (January 1997 - June 2013) to examine the association between metformin use and oncologic outcomes in 421 of diabetic patients undergoing radical cystectomy for bladder cancer and the authors concluded that metformin improves recurrence-free survival and bladder cancer-specific survival in diabetic patients undergoing radical cystectomy [25]. In addition, retrospective study conducted on 1117 patients with NMIBC at four institutions between 1996 and 2007 concluded that Patients with diabetic mellitus and NMIBC who do not take metformin seem to be at an increased risk of disease recurrence and progression [26]. Interestingly, a recent study demonstrated that metformin inhibits the proliferation of bladder cancer cells in vitro and in vivo [27]. Although, there are currently more than 100 ongoing or upcoming clinical studies assessing the role of metformin in the therapy cancer, only one ongoing clinical trial assessing the role of metformin (*NCT02360618*) (https://clinicaltrials.gov/ct2/show/NCT02360618) in the prevention of bladder cancer.

### Proposed mechanisms of anticarcinogenic effects of metformin

The potential beneficial effects of metformin against cancer are believed to be mediated mainly by one or more mechanisms that I will discuss further: (1) metformin induces growth inhibition, (2) metformin regulates insulin and glucose levels, (3) metformin induce cell death, (4) metformin potentiates the cytotoxicity of chemotherapeutic drugs, (5) metformin’s association with oxidative stress, DNA Damage Response (DDR), (6) immune and hypothalamic effects of metformin and (7) autophagy effects of metformin.

#### 1- Metformin induces growth inhibition

When Metformin is transported into the cells, it inhibits mitochondrial complex I (NADH: ubiquinone oxidoreductase) which consider as the first and largest enzyme of the respiratory chain and has a central role in cellular energy production through the coupling of NADH: ubiquinone electron transfer to proton translocation [28]. Thus, metformin has ability to decrease ATP synthesis [29]. As a result, the AMP: ATP ratio in the cell is increased, leading to energy stress and activation of AMPK (AMP-activated protein kinase), a primary metabolic sensor [30]. Hepatic AMPK activation can inhibit gluconeogenesis and activates glycolysis. In addition, AMPK activation can increase glucose consumption in muscle. Both of these consequences of metformin can diminish hepatic glucose output leading to lower systemic glucose and insulin levels, which could contribute to therapeutic effect in type II diabetes and impair malignant growth indirectly without requiring accumulation of metformin in the tumor (indirect effect of metformin on tumors) [31]. Furthermore, activation of AMPK leads to a cascade of downstream events resulting in mammalian target of rapamycin (mTOR pathway) down-regulation, which eventually induces protein synthesis arrest and growth inhibition [32, 33]. There are two different multiprotein complexes for mTOR, TORC1 and TORC2, which regulate protein synthesis necessary for cell growth, proliferation, angiogenesis, and other cellular endpoints [34]. Interestingly, mammalian target of rapamycin a member of the phosphatidylinositol 3-kinase (PI3K) cell survival pathway, plays an important role in the regulation of cell growth and proliferation by monitoring nutrient availability, cellular energy levels, oxygen levels and mitogenic signals [35]. Aberrant activation of the PI3K pathway has been widely implicated in many cancers, and increased activity of this pathway is often associated with resistance to cancer therapies [36].

#### Correlation of this pathway with bladder cancer

Several studies concluded that the AMPK pathway might influence both bladder cancer development and progression. A recent study by *Liu* et al. [37] reported that rhodiola rosea extract and salidroside inhibit the mTOR pathway and translational initiation via activation of AMPKα in UMUC-3 bladder cancer cells. In addition, PI3K and mTOR have prognostic/predictive value and represent valuable therapeutic targets in bladder cancer [38]. Metformin inhibits the proliferation of bladder cancer cells in vitro and in vivo through activation of AMPK and mTOR [19]. Moreover, metformin inhibits the growth of bladder cancer cells via indirect activation of AMPK [39, 40], which in turn suppresses the mTOR/p70 S6 kinase-1 (S6K1) pathway in 253 J and RT4 bladder cancer cell lines.

#### 2- Metformin regulates insulin and glucose levels

It is widely known that cancer cells express insulin as well as Insulin-like Growth Factor (IGF), Insulin-like Growth Factor Receptors (IGF-R) and that, besides its metabolic effect, IGF-R promotes proliferation and metastasis [41]. Cancer cells in particular have a constitutively high glucose uptake, independently of IGF-R activation [42]. However, hyperinsulinemia may promote tumor growth by various indirect mechanisms such as proliferation of epithelial tissue, increasing bioavailability of steroid sex hormones and serum levels of IGF, as well as disrupting the homeostasis of adipokines, which are cytokines selectively secreted by adipose tissue and thought to be implicated in cancer pathogenesis [43]. In addition, IGF activation promotes vascular smooth muscle cell proliferation and migration, promoting angiogenesis which could contribute to tumor growth [44].

Metformin activates AMPK which results in inhibition of gluconeogenesis in the liver, reducing insulin and glucose levels and increasing glucose uptake in skeletal muscle (a similar way as in metformin-treated diabetic patients) [45]. Metformin is thought to reduce ligand binding to insulin receptors; thus, metformin can indirectly down-regulate the insulin signaling pathway in tumours [46]. Moreover, metformin was shown to directly inhibit insulin induced malignant as well as benign cell growth in an AMPK/mTOR-dependent manner [47]. Recently, metformin was shown to exert a more direct effect on insulin signaling; by down-regulating a downstream target of the insulin receptors called Insulin Receptor Substrate-1 (IRS-1) [46]. The IRS-1 is a widely expressed protein that, in the presence of insulin, becomes phosphorylated by insulin receptors (or by IGF1-R) resulting in activation of downstream insulin-associated signaling pathways like PI3K-AKT/Protein Kinase B(PKB) and Ras-MAPK [48].

#### Correlation of this pathway with bladder cancer

Caloric intake appears to affect tumorigenesis through IGF. Higher caloric intake has been associated with an increased incidence of bladder cancer in American men less than 65 years of age [49]. In addition, the decreased caloric intake in mice slowed the growth of bladder tumors and this effect was reversed by IGF-1 administration [50]. Similarly, study found patients with elevated plasma IGF-1 levels to be three times more likely to develop bladder cancer [51]. Interestingly, study demonstrated that cells of human bladder cancer transfected with hsa-miR-96 inhibitor significantly reduced the growth of bladder cancer cells through reduction of mRNA and protein levels of IRS1 [52].

#### 3- Metformin induces cell death

Metformin was shown to promote cell death across multiple cell lines through both caspase-dependent and caspase-independent mechanisms [53]. It was shown that metformin decreases the expression of anti-apoptotic proteins B-cell lymphoma- 2 (BCL-2), B-cell lymphoma-extra-large (Bcl-Xl) and Myeloid cell leukemia-1 (Mcl-1), resulting to induction of the pro-apoptotic proteins, BCL2-Associated X Protein (BAX) and Bcl-2-Associated Death promoter (BAD) which lead to activation of caspases and apoptosis in Ovarian Cancer (OC) [54]. Metformin is also induced apoptosis by a caspase-independent mechanism involving the activation of PARP which results in nuclear translocation of AIF that leads to apoptosis [55]. More importantly, AMPK/mTOR-mediated decrease of suvivin in vivo which contributed in metformin-induced apoptosis of gastric cancer cell [56].

#### Correlation of this pathway with bladder cancer

A recent study concluded that nortriptyline has antitumor on human and mouse bladder cancer cells through induction of both intrinsic and extrinsic apoptosis. It increases the expression of Fas, FasL, FADD, Bax, Bak, and cleaved forms of caspase-3, caspase-8, caspase-9, and Poly (ADP-ribose) Polymerase (PARP). It also decreases the expression of Bcl-2, Bcl-xL, BH3 interacting domain death agonist, X-linked inhibitor of apoptosis protein, and survivin [57]. Survivin inhibits apoptosis by blocking activation of effector caspases in both extrinsic and intrinsic pathways of apoptosis. Moreover, survivin has also been indicated as a suitable target for developing specific therapy for local treatment of bladder cancer. Thus, survivin is a potentially significant protein with a crucial role in the diagnosis, prognosis and treatment of bladder cancer [58].

#### 4- Metformin potentiates the cytotoxicity of chemotherapeutic drugs

Metformin was shown to potentiate the cytotoxic effects of cisplatin in vitro [32] and in vivo studies; for instance metformin and cisplatin synergistically reduced size, proliferation and mitotic count of OC tumours in mice [59]. Metformin was shown also to potentiate the cytotoxic effects of carboplatin using OC cell lines and primary cultures from OC patients in advanced stages (III-IV) [60]. Moreover, in vitro studies showed that metformin acts synergistically with paclitaxel and potentiates its growth inhibitory effects in endometrial cancer [61]. Similarly, a study showed that the combination of metformin with Phenethyl Isothiocyanate (PEITC) increases growth inhibition and cytotoxicity in OC cell lines in a synergistic manner [62]. Interestingly, several studies have shown the advantages of combining metformin with standard cytotoxic drugs like cisplatin [59], taxol [63] and doxorubicin [64] or with molecular targeted agents such as gefitinib [65]. In addition, metformin showed a synergic effect when used in combination with 5-fluorouracil, in particular affecting CD133+ colorectal cancer cells viability in diabetic patients [66].

#### 5- Metformin’s association with oxidative stress, DNA damage and DNA damage response (DDR)

There are several mechanisms that get activated after DNA damage which occurred to avoid genomic instability; they are known as DNA Damage Response (DDR). One of the earliest DDRs is the activation of γH2AX as a result of DSB. This response occurs within minutes of the damage, thus making it a useful marker of DNA damage. The description of events involved in this activation in mammalian cells leading to γH2AX [67]. The earliest responding proteins are those of the Phosphatidylinositol 3-Kinase-Like family of Kinases (PIKK) including Ataxia Telangiectasia-Mutated (ATM), ATM- and Rad3-related (ATR) and the catalytic subunit of DNA-dependent Protein Kinase (DNA-PKc) [68].

Metformin exerts a protective effect from DNA damage as confirmed in several studies by reduction of DNA damaged signaling, γH2AX expression, γH2AX foci formation, ATM activation or Reactive Oxygen Species (ROS) levels by inhibition of mitochondrial complex I of the respiratory chain [68, 69]. Furthermore, inhibition of complex I compromises the electron flow in the electron transport chain, leading to reduced production of ROS by complexes I and III (mitochondrial ROS producers) [70].

#### Correlation of this pathway with bladder cancer

Mechanisms governing treatment-induced DNA damage are both central to and predictive of bladder cancer cell treatment sensitivity and exemplify a link between DNA damage resistance and both treatment response and tumour aggression [71]. On the other hand, study conducted by *Camargo* et al.*,* indicated that no relationship was observed between the amount of DNA damage and the level of hMLH1 (a gene involved in the mismatch repair pathway) and RASSF1 (a tumor suppressor gene) in bladder cancer cells treated with cisplatin and gemcitabine. They also confirmed other alternative pathways might be involved in cisplatin and gemcitabine genotoxicity in bladder cancer cells [72].

#### 6- Immune and hypothalamic effects of metformin

The concept of immune-modulating effects of metformin was originally proposed in the 1950s by the Philippine physician Garcia [73]. A recent study suggested that metformin can increase the number memory CD8 T cells in wild type mice, and in consequence significantly improve the efficacy of an experimental anticancer vaccine through increased fatty acid oxidation [74]. In addition, study by *Ropelle* et al., have shown that hypothalamic AMPK activation in response to metformin reverses cancer anorexia in tumor bearing rats through inhibiting the production of proinflammatory molecules and controlling neuropeptide expression in the hypothalamus [75].

#### Correlation of this pathway with bladder cancer

A high fraction of adaptive immune markers CD3 (the whole T cell population) and CD8 (T effector cells) in bladder cancer indicated a poor prognosis, thereby emphasising the important role that Tregs play in the suppression of the anti-tumour immune response [76].

#### 7- Autophagy effects of metformin

Autophagy is a self-degradative process that is important for balancing sources of energy at critical times in development and in response to nutrient stress. It also plays a housekeeping role in removing misfolded or aggregated proteins, clearing damaged organelles, such as mitochondria, endoplasmic reticulum and peroxisomes, as well as eliminating intracellular pathogens. Thus, it is generally thought of as a survival mechanism, although its deregulation has been linked to non-apoptotic cell death [77].

Metformin can enhance autophagy, as AMPK activation is known to upregulate autophagic activity through direct phosphorylation of unc- 51-like kinase and Beclin 1, key molecules involved in the initiation of autophagy [78]. The activation of AMPK by metformin proposes the possibility that improvement in metabolic profiles by metformin might be related to autophagy induction through AMPK activation. In addition, metformin has been shown to enhance disposal of accumulated autophagic vacuoles in β-cells [79]. It has been reported also to enhance autophagic activity in cardiac tissue by facilitating dissociation of the Bcl-2-Beclin 1 complex through AMPK activation [80] and ameliorating ultra-structural abnormalities associated with diabetes in an animal model of diabetic cardiomyopathy [81]. Interestingly, a recent study reported amelioration of hepatic steatosis by metformin through autophagy activation via sirtuin 1 pathway rather than AMPK pathway [82]. The sirtuin 1 could influence autophagy directly via its deacetylation of key components of the autophagy induction network, such as the products of Autophagy genes (Atg) 5, 7, and 8 [83].

#### Correlation of this pathway with bladder cancer

A recent study showed that metformin suppresses endometrial cancer cell growth through cell cycle arrest and concomitant apoptosis and autophagy. These results indicated that the anti-proliferative effects and apoptosis caused by metformin are partially or completely dependent on autophagy [84]. Similarly, several studies have indicated that Troglitazone affects both cell growth and differentiation progress and has an inhibitory effect on urinary cancer cells by activation of autophagy concurrent with the activation of the AMPK signaling pathway [85]. Moreover, further studies have shown that gartanin treatment of bladder cancer cell lines T24 and RT4 resulted in a marked induction of autophagy and apoptosis [86].

## Conclusion

There is lacking of ongoing or upcoming clinical studies assessing the role of metformin in the therapy of bladder cancer. Recently, metformin can inhibit the proliferation of bladder cancer cells in vitro and in vivo. Moreover, DM and obesity are considered as risk factors for bladder cancer. Metformin, a first-line oral anti-diabetic, has been demonstrated to prevent cancer and reduce cancer mortality among diabetic patients in several observational studies. Furthermore, metformin is commonly used off-label for metabolic syndrome and treat weight gain. Interestingly, metformin was shown to potentiate the cytotoxic effects of numerous chemotherapeutic drugs which common to be used in the treatment of bladder cancer such as cisplatin, carboplatin, paclitaxel, PEITC, doxorubicin, gefitinib and 5-fluorouracil. This is the first review shed light on this direction to open the door towards further researches and clinical studies on the application of metformin as adjuvant therapy for bladder cancer taken the advantages and proposed mechanisms of anti-carcinogenic effects of metformin which summarized in this review to confirm or not novel usage of metformin through its ongoing journey across cancer therapy.

## Abbreviations

AIF: Apoptosis-inducing factor
Atg: Autophagy genes
ATM: Ataxia telangiectasia-mutated
ATR: ATM- and rad3-related
BAD: Bcl-2-associated death promoter
BCL-2: B-cell lymphoma- 2
Bcl-Xl: B-cell lymphoma-extra-large
BMI: Body mass index
CRC: Colorectal cancer carcinogenesis
DDR: DNA damage and DNA damage response
DM: Diabetes mellitus
DNA-PKc: DNA-dependent protein kinase
DSB: Double-strand breaks
IARC: International agency for research on cancer
IEO: European institute of oncology
IGF: Insulin-like growth factor
IGF-R: Insulin-like growth factor receptors
IRS-1: Insulin receptor substrate-1
Mcl-1: Myeloid cell leukemia-1
MIBC: Muscle invasive bladder cancer
NMIBC: Non-muscle-invasive bladder cancer
OC: Ovarian cancer
PARP: poly (ADP-ribose) polymerase
PEITC: Phenethyl Isothiocyanate
PI3K: Phosphatidylinositol 3-kinase
PIKK: Phosphatidylinositol 3-kinase-like family of kinases
RCTs: Randomized controlled trials
ROS: Reactive oxygen species
