# Correction to: New insight for metformin against bladder cancer

**DOI:** 10.1186/s41021-018-0105-4

**Published:** 2018-07-24

**Authors:** Amr Ahmed EL-Arabey

**Affiliations:** 1 Pharmacology and Toxicology Department, Faculty of Pharmacy, Al-Azhar University, Nasr City, Cairo Egypt; 2 CAS-TWAS Fellowship at University of Science and Technology of China (USTC), Hefei, 23027 China

## Correction

The author noted a few errors in the original article [1] need correction. The correct content is below.

1. The 3rd paragraph in background should be removed due to duplication.
2. In page 2 under *Anti-carcinogenic effects of metformin*, line 15, the ‘in vitro and in vitro’ should be ‘in vitro and in vivo’.
